# Integrative phosphoproteomic analysis identifies functional roles of TRPM7 phosphosites in oncogenesis

**DOI:** 10.3389/fbinf.2026.1757645

**Published:** 2026-06-01

**Authors:** Akhina Palollathil, Althaf Mahin, Athira Perunelly Gopalakrishnan, Alimath Sambreena, Prathik Basthikoppa Shivamurthy, Rajesh Raju

**Affiliations:** Centre for Integrative Omics Data Science (CIODS), Yenepoya (Deemed to be University), Mangalore, Karnataka, India

**Keywords:** ion channel, mass spectrometry, phosphoproteomics, phosphorylation, transient receptor potential melastatin 7, TRPM7, kinase, post-translational modification

## Abstract

**Introduction:**

Transient Receptor Potential Melastatin 7 (TRPM7) is a ‘chanzyme’ with dual functions, acting both as a channel for divalent ions and as a serine/threonine kinase. Overexpression of TRPM7 has been linked to the development of various diseases, particularly cancers, making it a promising molecular target. Despite its relevance in oncogenesis, the phospho-regulatory network of TRPM7 remains largely unexplored, with limited evidence on its upstream kinases, downstream substrates, and site-specific phospho-regulated functions.

**Methods:**

To address this knowledge gap, we employed a co-differential detection-based strategy to analyse publicly available phosphoproteomics datasets.

**Results:**

Through the analysis of 569 phosphoproteomics profiling datasets and 116 differential abundance datasets, we identified 55 and 38 Class I phosphosites in TRPM7, of which 13 have not been previously reported. Among the Class I phosphosites, S1504, S1255, S1513, S1477, and S1387 emerged as the predominant phosphosites in TRPM7. Furthermore, all known interactors and substrates of TRPM7 were associated with broad cellular functions such as protein phosphorylation, chromatin remodeling, transcriptional regulation, intracellular signal transduction, DNA damage response, and apoptosis, whereas the co-differentially regulated interactors and substrates of TRPM7 were associated with more specialized functions, including positive regulation of stem cell population maintenance, regulation of mRNA splicing via the spliceosome, and regulation of the G2/M phase transition of the cell cycle. Finally, we identified potential upstream kinases for TRPM7, including PRKCD, CLK2, STK39, PKN2, MAST3, PRKD3, and MAP4K4.

**Discussion:**

These findings provide a comprehensive resource of TRPM7 phosphosites, their potential regulatory kinases, and associated biological functions, laying the groundwork for future mechanistic and therapeutic studies.

## 1 Introduction

Transient Receptor Potential Melastatin 7 (TRPM7) belongs to the Transient Receptor Potential (TRP) ion channel family and is a member of the Melastatin subfamily. TRPM7 is one among the eight members (TRPM1-8) in the Melastatin subfamily that mediate cellular responses to diverse environmental and physiological stimuli, including hormones, pH changes, and temperature variations (Curry et al., 2024). TRPM7 was initially discovered by Runnels et al. (2001) through homology-based screening for members of the TRP channel family and is distinguished as a chanzyme, exhibiting dual functionality as both a cation-permeable ion channel and a serine/threonine kinase (Runnels et al., 2001). Structurally, TRPM7 contains six transmembrane helices that form a channel pore permeable to divalent cations, including Mg^2+^, Ca^2+^, and Zn^2+^. This region is followed by a conserved TRP domain that plays a critical role in tetramerization (Runnels et al., 2001; Monteilh-Zoller et al., 2003). The C-terminal region of TRPM7 harbours an α-kinase domain that possesses intrinsic catalytic activity and can phosphorylate multiple downstream substrates, including ANXA1 (annexin A1), MYH9 (Myosin Heavy Chain 9), and PLCG2 (Phospholipase C Gamma 2) (Dorovkov et al., 2011; Clark et al., 2008a; Deaso et al., 2012). ANXA1 is a calcium-regulated protein with anti-inflammatory activity through inhibition of phospholipase A2 and pro-inflammatory activity through induction of cytokine expression (Shao et al., 2019). MYH9 is a non-muscle myosin involved in the regulation of cytoskeletal organization, cell division, and cell motility (Emami et al., 2026). PLCG2 is a calcium-dependent transmembrane signaling enzyme with a vital role in autoinflammation and immune homeostasis (Jackson et al., 2021). Caspase-mediated cleavage of TRPM7 separates the kinase domain from the ion-conducting channel, resulting in a hyperactive channel while the detached kinase remains catalytically functional and can translocate to the nucleus to modulate gene expression (Des et al., 2012).

TRPM7 channel activity is critical for the regulation of divalent cations Mg^2+^, Zn^2+^, and Ca^2+^ homeostasis, which are essential for cell proliferation, survival, migration, and cytoskeletal organization (Resende et al., 2013; Rakshit et al., 2023; Tsai et al., 2015). TRPM7 engages in cross-talk with key intracellular signaling pathways, including MAPK, PI3K/AKT, and JAK/STAT pathways, thereby coupling ion homeostasis to diverse downstream cellular processes (Zou et al., 2019). Dysregulation of TRPM7 has been associated with several human diseases. In cardiovascular and neurological disorders, abnormal TRPM7 activity contributes to ischemic neuronal death, cardiac arrhythmias, and fibrosis (Jimenez et al., 2020; Hu et al., 2021; Sun, 2017).

Elevated expression of TRPM7 has been reported in various cancers, including pancreatic, ovarian, glioblastoma, head and neck, lung, prostate, liver, renal, breast, and gastric cancers, whereas downregulation or functional inhibition can impair tumour growth and induce apoptosis (Koles et al., 2024; Yee, 2017). The involvement of TRPM7 in cancer was first reported by Dai et al. (2007), who found that the Thr1482Ile polymorphism was associated with an increased risk of colorectal adenomas or hyperplastic polyps, particularly among individuals with high dietary intake of Ca2+/Mg2+ (Dai et al., 2007). Subsequently, several studies demonstrated that elevated TRPM7 expression is associated with enhanced cancer cell proliferation, migration, invasion, and metastatic potential across multiple cancer types (Koles et al., 2024). TRPM7 promotes cancer progression through diverse mechanisms involving ion homeostasis, cytoskeletal regulation, and extracellular matrix remodelling. In pancreatic cancer, TRPM7 promotes Mg2+ mediated cellular motility and stimulates invasive activity through activation of the Hsp90α/uPA/MMP-2 proteolytic axis (Rybarczyk et al., 2012; Rybarczyk et al., 2017). Increased mRNA level expression of TRPM7 was associated with shorter recurrence-free survival and distant metastasis-free survival in breast cancer. Knockdown of TRPM7 attenuated the metastatic ability of breast cancer by increasing the focal adhesions through regulating myosin II–based cellular tension (Middelbeek et al., 2012). In addition, TRPM7 promotes cancer metastasis through the activation of multiple cellular signaling pathways, including PI3K/AKT, MAPK, SRC, and JNK signaling (Lee et al., 2020; Ha et al., 2018; Meng et al., 2013). TRPM7 promote the proliferation, growth, stem cell stemness, and migration of glioblastoma cells by inducing the expression of ALDH1, CD133, and FOSL1 (Wan et al., 2020; Guo et al., 2022). Elevated TRPM7 expression in head and neck cancers, including nasopharyngeal, laryngeal, and hypopharyngeal carcinomas, has been associated with poor prognosis, whereas lower TRPM7 expression correlates with improved patient survival (Chen et al., 2015; Qin et al., 2016; Chen et al., 2022). Studies in gastric cancer have reported that inhibition of TRPM7 channel activity using plant-derived extracts suppresses the growth and survival of gastric cancer cells (Lee et al., 2013; Lim et al., 2013). These reports collectively highlight the multifaceted role of TRPM7 in tumor progression and metastasis. Although previous studies have demonstrated the pivotal role of TRPM7 in cancer, a comprehensive, site-specific phosphorylation landscape of TRPM7 remains underexplored, with limited information available on the functional roles of individual phosphosites. As phosphoproteomic datasets remain only partially interpreted, systematic reanalysis of publicly available data offers an opportunity to extract deeper functional insights and uncover understudied regulatory phosphosites. In this study, we employed a phosphosite co-detection-based *in silico* strategy to identify functionally relevant phosphorylation events in TRPM7 from large-scale phosphoproteomic datasets. By integrating co-detected phosphoprotein patterns across diverse biological conditions, we identified predominant TRPM7 phosphosites, predicted their potential upstream kinases, and inferred biological functions for currently uncharacterized sites based on the cellular roles of co-detected phosphosites on other proteins. This comprehensive approach not only provides deeper insights into TRPM7 phosphorylation but also lays the groundwork for future studies investigating its disease associations, functional roles, and potential as a therapeutic target across diverse pathological contexts.

## 2 Materials and methods

### 2.1 Systematic analysis of phosphoproteomic data to prioritise TRPM7 phosphosites

The phosphoproteomics datasets mining and data analysis strategy employed in this study were adopted from our previously published works (Raghu et al., 2025; Chakraborty et al., 2025; Sanjeev et al., 2024; Mahin et al., 2025). Briefly, this study included original research articles indexed in PubMed that reported global, high-throughput phosphoproteomic analyses performed under human cell line-based experimental conditions and provided phosphorylation site-level data. Studies were excluded if they were review articles, conducted exclusively in plants, mice, rats, or other non-human model organisms, or lacked accessible and extractable phosphorylation site information. The Class-I phosphosites (localization probability ≥75% or A score >13) reported from various phosphoproteomics studies were manually curated, and protein identifiers were standardized to HUGO Gene Nomenclature Committee (HGNC) gene symbols and UniProt accessions to ensure uniform mapping. We also recorded additional information associated with each dataset, including phosphopeptide enrichment protocols, cell lines used, and the corresponding biological and experimental conditions, to facilitate downstream analyses. The selected datasets encompassed both phosphoproteomic profiling and differential abundance datasets. Differential phosphopeptides were classified based on fold-change thresholds of ≥1.3 for increased abundance and ≤0.76 for decreased abundance, and p-value <0.05 for statistical significance.

In this study, a “dataset” refers to a single phosphoproteomic comparison corresponding to one specific experimental condition within that publication. Further, the detection of each TRPM7 phosphosite was assessed across both qualitative and quantitative phosphoproteomics datasets, and sites were ranked according to how consistently they were detected. Some phosphosites appeared only in a few, whereas others were repeatedly identified across multiple experimental conditions. Sites observed in the majority of datasets were designated as predominant and prioritised for further analysis, while rarely detected Class-I sites were excluded from downstream investigations.

### 2.2 Identification and statistical assessment of co-differential detection patterns between TRPM7 and other protein phosphosites

To understand the regulatory landscape of TRPM7, we developed a systematic co-differential detection analysis framework to identify phosphosites on other proteins (PsOPs) that exhibit coordinated phosphorylation changes with the predominant TRPM7 sites across diverse cellular perturbations. PsOPs detected in the differential abundance datasets were classified into four regulatory categories (UU, DD, UD, and DU) based on their phosphorylation changes in relation to the predominant TRPM7 phosphosites. The UU category included PsOPs that exhibited increased phosphorylation concomitant with the hyperphosphorylation of the predominant TRPM7 site. Likewise, PsOPs that were hypophosphorylated when the TRPM7 predominant site was also hypophosphorylated were grouped under the DD category. In contrast, PsOPs showing hypophosphorylation despite the hyperphosphorylation of the TRPM7 site were assigned to the UD group, whereas those displaying hyperphosphorylation when the TRPM7 site was hypophosphorylated were categorized as DU. Furthermore, phosphosites classified as UU or DD were collectively grouped under the UUDD category, representing positive co-differential detection with the predominant TRPM7 site. Conversely, phosphosites falling into the UD or DU categories were grouped as UDDU, indicative of negative co-differential detection. Subsequently, we computed the number of quantitative phosphoproteomic datasets in which each PsOPs exhibited a relationship (UUDD or UDDU) with the predominant TRPM7 phosphosite. Phosphosites exhibiting significant positive or negative co-differential detection with the predominant TRPM7 sites were then filtered and compiled separately for downstream analyses (Sanjeev et al., 2024; Mahin et al., 2025).

To assess the significance and likelihood of the coregulation patterns, a one-sided Fisher’s exact test (FET) was performed. We applied Fisher’s exact test following the analysis framework established by Li et al. (2017) to evaluate the statistical significance of phosphosite co-occurrence (Li et al., 2017). The co-differentially detected PsOPs with an FET p-value less than 0.05 were considered statistically significant. Additionally, high-confidence co-differentially detection patterns were subsequently identified by calculating a co-differential detection ratio. For positive co-differentially detection, the ratio was defined as ∑(nUUDD)/∑(nUDDU), while for negative co-differentially detection, it was calculated as ∑(nUDDU)/∑(nUUDD) (zero values were considered as 1 for the ratio). The ratio reflects the relative prevalence of positive co-detection over negative co-detection, and *vice versa*, across the analysed datasets. The PsOPs with a co-differential detection ratio exceeding 10% of the total differential detection frequency of the predominant TRPM7 sites were considered high-confidence co-differentially detected pairs. To minimize potential bias due to phosphosites identified only from one or two studies, or the over-representation of any multi-temporal datasets of a single stimulus type, additional filtering was applied based on PubMed IDs (PMIDs) and experimental codes (studies using the same stimuli grouped under a single experimental code). For each phosphosite, the experimental code confidence was computed as UUDD EXP Code − UDDU EXP for positive co-differential detection, and UDDU EXP Code − UUDD EXP Code for negative co-differential detection. The PMID confidence was determined by the number of unique studies supporting a given co-differential detection pattern. Only phosphosites meeting the thresholds of ≥3 for experimental code confidence and PMID confidence were retained as highly significant positive or negative co-differential detection patterns (Raghu et al., 2025; Chakraborty et al., 2025).

### 2.3 Co-occurrence analysis for phosphosites within TRPM7

Li et al. (2017) demonstrated that co-occurring phosphorylation sites within a protein are often functionally linked and subject to similar regulatory mechanisms (Li et al., 2017). To identify co-occurring TRPM7 phosphosites, all sites identified from differential abundance datasets were organized according to experimental conditions. The frequencies of positive (n_UUDD) and negative (n_UDDU) co-occurrence among these sites were then calculated following the methodology described above.

### 2.4 Compilation of phosphosite-specific interactors and kinases of TRPM7

The experimentally known, direct (binary) and indirect (complex-based) protein interactors of TRPM7 were compiled from various databases, including BioGRID (Oughtred et al., 2019), IntAct (Del Toro et al., 2022), BIND (Bader et al., 2003), HPRD (Keshava Prasad et al., 2009), and PhosphoPOINT (Yang et al., 2008).

Experimentally confirmed kinases targeting specific TRPM7 phosphosites were retrieved from PhosphoSitePlus (Hornbeck et al., 2012), Phospho.ELM 9.0 (Dinkel et al., 2011), and RegPhos 2.0 (Huang et al., 2014). Predicted kinases were obtained from NetworKIN (retrieved on 04.01.2023) (Linding et al., 2008), AKID (retrieved on 24.05.2023) (Parca et al., 2019), and iKiP-DB (Mari et al., 2022). Additionally, kinases and substrates predicted for TRPM7 by Johnson et al. (2023), with a confidence cutoff of 90%, were also included (Johnson et al., 2023).

### 2.5 Functional enrichment analysis of co-differentially detected proteins

To illustrate the distribution of kinases, the kinome map was generated using the online tool, KinMap (Eid et al., 2017). Site-specific biological functions of these co-differentially detected proteins were obtained from PhosphoSitePlus (Hornbeck et al., 2012). Additionally, to explore the biological functions of all known interactors and substrates, as well as co-differentially detected interactors and substrates of TRPM7, we performed Gene Ontology enrichment analysis using the Enrichr platform (Chen et al., 2013).

### 2.6 Data visualization

The lollipop plot illustrating TRPM7 phosphosites was generated in RStudio using the Bioconductor package trackViewer (Ou and Zhu, 2019). Frequencies of co-differentially regulated phosphosites on other proteins (PsOPs) across the differential abundance datasets were visualized using Matplotlib in Python. Co-occurring TRPM7 phosphosites were represented as a correlation matrix generated with the Seaborn Python package. The interaction network was constructed and visualized using Cytoscape (Shannon et al., 2003). The Sankey diagram representing the biological process was generated using the RAWGraphs (https://www.rawgraphs.io/).

## 3 Results

### 3.1 Frequently perturbed phosphosites in TRPM7 identified from phosphoproteomics datasets

**FIGURE 1:**
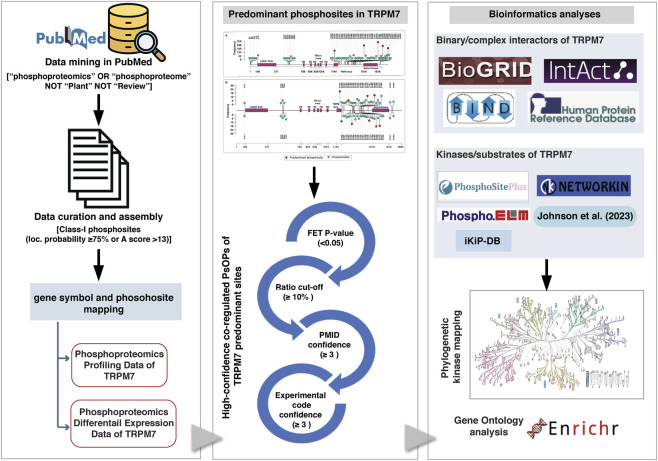
Schematic overview of the data assembly and analytical pipeline used to identify TRPM7 phosphosites and their co-differentially detected phosphosites in other proteins (PsOPs). Integration of phosphoproteomics datasets derived from human cell lines from PubMed. Mapping of gene symbol and phosphosite to the latest UniProt release. Predominant TRPM7 phosphosites were identified based on detection frequency across profiling and differential expression datasets. Co-differential detection between TRPM7 phosphosites and PsOPs was assessed using a one-sided Fisher’s exact test. High-confidence PsOPs were selected using stringent filtering criteria and further analyzed for their association with known and predicted interactors, upstream kinases, and substrates.

The large-scale phosphoproteomics datasets serve as a rich resource for understanding the complex regulatory networks that govern cellular signaling and protein function. The integration and reanalysis of publicly accessible phosphoproteomic datasets hold immense promise for advancing systems-level insights into cellular regulation and disease mechanisms (Schweiger and Linial, 2010). To identify the most frequently detected (predominant) phosphosites in TRPM7, mass spectrometry-based phosphoproteomic studies were retrieved from PubMed. The potential functional roles of predominant TRPM7 phosphosites were inferred based on phosphosites on other proteins positively or negatively co-differentially detected with TRPM7 (Figure 1).

**FIGURE 2:**
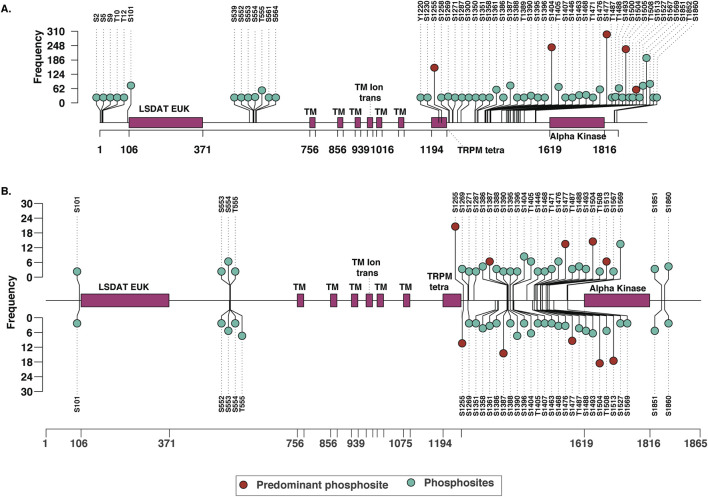
Frequency of phosphorylation on serine (S), threonine (T), and tyrosine (Y) residues in TRPM7, as detected from phosphoproteomics studies. **(A)** Class-I phosphosites in TRPM7 identified from 569 phosphoproteomics profiling datasets. **(B)** Class-I phosphosites in TRPM7 identified from 116 differential expression datasets.

Of the 3825 phosphoproteomic studies retrieved, 685 datasets were associated with TRPM7 phosphorylation, comprising 569 phosphoproteomics profiling datasets and 116 datasets describing differential phosphorylation under various biological and experimental conditions (Supplementary Tables S1, S2). Systematic analysis of these datasets identified 55 Class I phosphosites in TRPM7 from the profiling data and 38 Class I phosphosites from the differential datasets (Figures 2A,B). According to the PhosphoSitePlus database, TRPM7 is extensively phosphorylated, with 87 reported phosphosites distributed across serine (56 sites), threonine (24 sites), tyrosine (2 sites), and an additional 5 sites occurring on other residues. When comparing the phosphosites identified for TRPM7 from our profiling datasets with those reported in PhosphoSitePlus, 42 of the 55 detected sites were already documented in the database. Interestingly, we also detected 13 additional phosphosites (S2, T1852, Y1220, T1389, T1487, S553, S1351, S1350, S1388, S564, S561, S539, and S1300) that are not currently listed in PhosphoSitePlus. Among these newly identified sites, four (S1351, S553, S1388, and T1487) were also observed in the differential abundance datasets (Figures 2A,B). Most of these additional sites were located in the Ser/Thr-rich regions (a.a. 1300–1487) positioned just upstream of the kinase domain.

In the profiling datasets, Class-1 phosphosites such as S1477, S1404, S1504, S1569, and S1255 were identified as predominant, being detected in 304, 248, 240, 202, and 160 datasets, respectively. Likewise, in the differential abundance datasets, S1504, S1255, S1513, S1477, and S1387 emerged as predominant phosphosites, supported by 34, 32, 25, 24, and 22 datasets, respectively. Further, we prioritized phosphosites showing the highest detection across both profiling and differential abundance datasets for downstream analysis.

### 3.2 Co-occurrence of phosphosites within TRPM7

**FIGURE 3:**
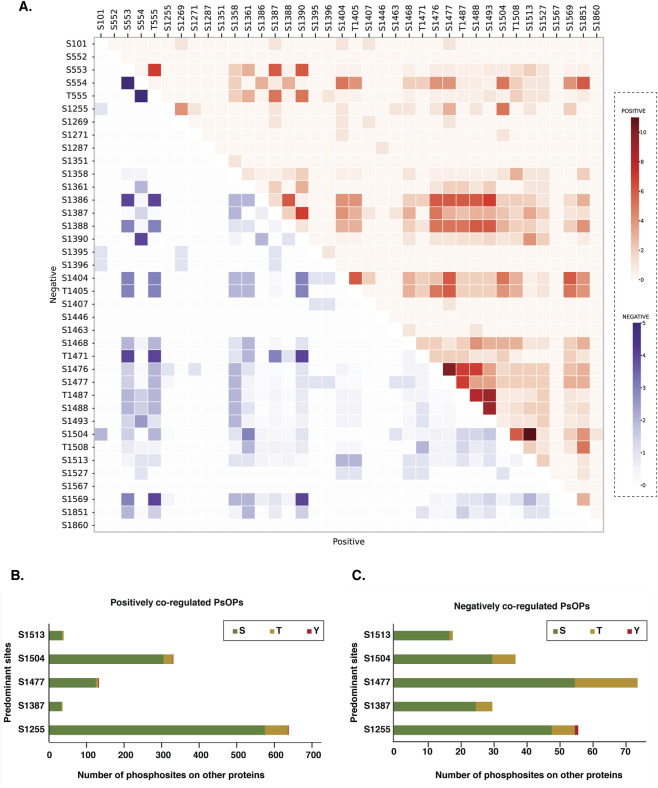
Co-occurring phosphosites in TRPM7 identified from phosphoproteomics datasets. **(A)** TRPM7 phosphosites showing positive and negative co-occurrence patterns from differentially abundant phosphoproteomics data. Positive co-occurrence is depicted in red, while negative co-occurrence is shown in blue, with colour intensity reflecting the strength of the association. **(B)** The bar plot illustrates the number of serine (S), threonine (T), and tyrosine (Y) phosphorylations on other proteins that show positive or **(C)** negative co-differential detection with the predominant phosphosites in TRPM7.

Previous studies have shown that functionally linked phosphosites within a protein often occur simultaneously, and this coordinated phosphorylation is crucial for the protein’s proper function. Identifying such co-occurring phosphosites will help to gain a comprehensive understanding of protein functions (Li et al., 2017). In some instances, these co-occurring sites may be phosphorylated by the same or related kinases (Schweiger and Linial, 2010). We conducted a co-occurrence analysis across phosphoproteomic datasets, identifying instances where multiple sites were detected together within the TRPM7. The frequency of these co-occurring phosphosites was then quantified and ranked based on their positive or negative association patterns. The analysis revealed multiple phosphosites that tend to occur simultaneously within TRPM7 (Figure 3; Supplementary Table S3). For instance, the predominant phosphosite S1255 frequently co-occurred with other sites in TRPM7, such as S1269, S1477, and S1504, indicating coordinated phosphorylation events. Likewise, S1387 was found to co-occur with S1504, S1513, S1390, and S1477. While S1477 co-occurred with T1487, T1405, and S1476. In addition, S1504 was detected alongside S1513. Collectively, these predominant sites exhibited strong positive co-differential detection, as supported by their highest frequency of simultaneous detection in UUDD datasets than in UDDU datasets.

### 3.3 Phosphosites on other proteins exhibiting co-differential regulation with TRPM7 predominant sites

As evidenced from the PhosphoSitePlus database, the biological roles of TRPM7 phosphosites remain uncharacterized, with no functional annotations currently available for these modification sites. This gap in knowledge highlights the importance of further investigation to elucidate the regulatory mechanisms of TRPM7 phosphosites in various biological functions and cellular signaling pathways. Since most proteins do not function in isolation, those that exhibit coordinated changes in abundance and physically interact are likely to participate in related biological processes (Siwiak and Zielenkiewicz, 2015; Piovesan et al., 2015). Therefore, we employed a co-differential detection-based strategy to infer the potential phosphosite-specific functions of TRPM7.

**FIGURE 4:**
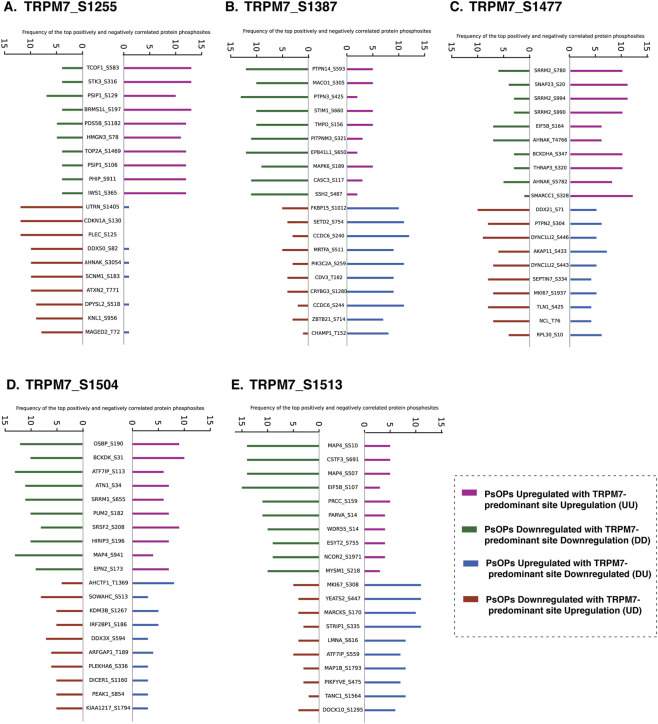
The PsOPs that are highly positively and negatively co-differentially regulated with the predominant phosphosites on TRPM7. PsOPs that are positively and negatively co-differentially regulated with the TRPM7 **(A)** S1255, **(B)** S1387, **(C)** S1477, **(D)** S1504, **(E)** S1513.

To investigate the co-differential regulation of phosphosites on other proteins (PsOPs) with the predominant TRPM7 sites (S1504, S1255, S1513, S1477, and S1387), differential abundance datasets were categorized into four groups: (1) both PsOPs and the TRPM7 site showed increased abundance, (2) both showed decreased abundance, (3) PsOPs showed increased abundance while the TRPM7 site showed decreased abundance, and (4) PsOPs showed decreased abundance while the TRPM7 site showed increased abundance. Groups 1 and 2 were considered positive co-differential detection, whereas groups 3 and 4 were considered negative co-differential detection, as they show similar trends in abundance patterns. Significantly co-differentially detected phosphosite pairs were identified using a one-sided FET p-value threshold of <0.05 and further filtered based on supporting evidence from at least three independent studies and experimental conditions (Supplementary Tables S4–S5). After this filtering, 638, 36, 133, 331, and 39 PsOPs were found to be positively co-differentially detected with the predominant TRPM7 sites S1255, S1387, S1477, S1504, and S1513, respectively (Figure 3B; Supplementary Tables S4A–S4E). Likewise, 56, 30, 73, 37, and 18 PsOPs showed negative co-differential detection with predominant sites (Figure 3C; Supplementary Tables S5A–S5E). Among the top positively co-differentially detected PsOPs, TCOF1_S583 was strongly associated with S1255; PTPN14_S593 with S1387; SRRM2_S780 with S1477; OSBP_S190 with S1504; and MAP4_S510 with S1513. In contrast, the most strongly negatively co-differentially detected partners included UTRN_S1405 for S1255; FKBP15_S1012 for S1387; DDX21_S71 for S1477; AHCTF1_T1369 for S1504; and MKI67_S308 for S1513 (Figures 4A–E).

Additionally, we evaluated the overall phosphorylation patterns of proteins positively and negatively co-differentially detected with TRPM7 phosphosites. The analysis revealed that proteins such as SRRM2, SRRM1, BCLAF1, IWS1, CDK12, and TJP1 exhibited the highest numbers of overall phosphosites among the co-detected proteins associated with TRPM7 phosphorylation. A total of 57 phosphorylation sites in SRRM2 were positively co-differentially detected with TRPM7 phosphosites, while no negatively co-differentially detected SRRM2 phosphosites were identified across the analyzed datasets. Likewise, 11 and 10 phosphosites in SRRM1 and IWS1, respectively, exhibited positive co-differential detection patterns with TRPM7 phosphosites. In BCLAF1, nine phosphosites were found in positive co-differential data and one site in negative co-differential data. The details on the overall phosphorylation patterns of co-detected proteins are provided in Supplementary Tables 4F, 5F

### 3.4 Identification of co-differentially detected interactors and substrates of TRPM7

The biological functions of a protein are tightly regulated by PTMs through modulating their activation and interactions. Among well-studied PTMs, phosphorylation plays a crucial role in either activating or inhibiting protein function. To gain deeper insights into the biological roles of TRPM7, we assessed the known binary and complex interactors of TRPM7 among the co-differentially detected data. Binary interactors refer to proteins that have direct physical interactions with TRPM7, whereas complex interactors refer to proteins that are part of the same multi-protein complex but may not interact directly with TRPM7. For this purpose, the known interacting partners of TRPM7 were retrieved from multiple protein-protein interaction databases. The analysis identified 9 binary interactors and 174 complex interactors exhibiting positive coordination with predominant phosphosites. In contrast, 3 binary interactors and 28 complex interactors showed negative co-differential detection (Supplementary Tables S4, 5).

As only a limited number of experimentally validated substrates have been identified for TRPM7, we employed a combined strategy incorporating computational kinase-substrate prediction platforms along with candidate substrates reported by Johnson et al. (2023) to infer its potential substrate network (Johnson et al., 2023). From the positively co-differentially detected subset, 40 substrates corresponding to TRPM7_S1255 were obtained from Johnson et al. (2023) and three additional substrates from prediction tools. Likewise, for TRPM7_S1387, TRPM7_S1477, and TRPM7_S1504, we identified three, six, and 22 substrates, respectively, from Johnson et al. (2023). In contrast, five, three, six, three, and one substrates were negatively co-differentially detected with TRPM7 predominant sites S1255, S1387, S1477, S1504, and S1513, respectively (Supplementary Tables S6A–B). All negatively co-differentially detected substrates were sourced from Johnson et al. (2023), except for one substrate associated with TRPM7_S1504, which was obtained from the prediction tools.

**FIGURE 5:**
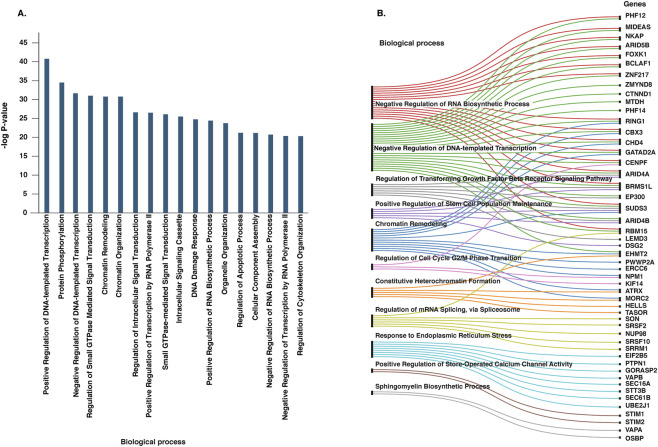
Biological processes regulated by interactors and substrates of TRPM7. **(A)** Bar plot illustrating the highly enriched biological processes for all known interactors and substrates of TRPM7. **(B)** Biological processes enriched for co-differentially phosphorylated interactors and substrates of TRPM7.

To obtain a broader understanding of the biological processes associated with TRPM7, we performed Gene Ontology enrichment analysis of all known interactors and substrates of TRPM7 and compared these results with the enrichment profiles obtained from interactors/substrates containing phosphosites that were co-differentially detected with TRPM7 phosphosites. The biological process enriched for all known interactors and substrates revealed broad cellular functions associated with TRPM7, including positive regulation of DNA-templated transcription, protein phosphorylation, chromatin remodeling, regulation of intracellular signal transduction, and DNA damage response (Figure 5A and Supplementary Table S7A). In contrast, a more specific set of biological processes was enriched for interactors and substrates containing phosphosites co-differentially detected with TRPM7 phosphosites, such as positive regulation of stem cell population maintenance, regulation of mRNA splicing, via spliceosome, response to endoplasmic reticulum stress, regulation of cell cycle G2/M phase transition, and regulation of transforming growth factor beta receptor signaling pathway (Figure 5B; Supplementary Table S7B). Comparative analysis demonstrated that focusing on interactors and substrates co-differentially phosphorylated with TRPM7 provides greater functional specificity and biological insight.

### 3.5 Identification of potential upstream kinases of TRPM7

**FIGURE 6:**
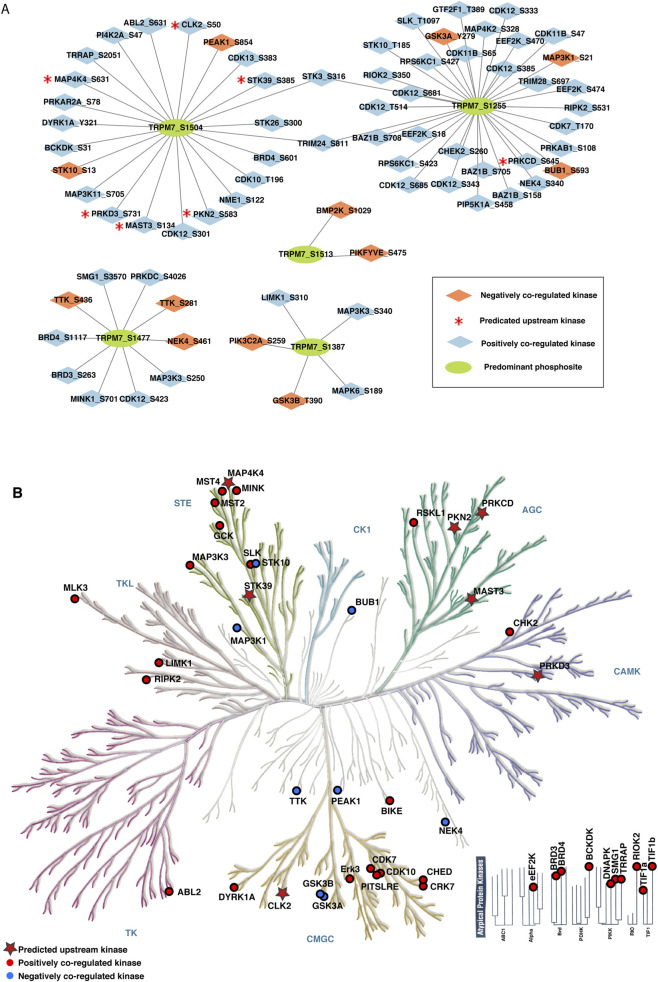
Co-differentially expressed kinases of TRPM7 predominant sites. **(A)** Network illustrates the positively and negatively regulated potential kinases of TRPM7. **(B)** The kinome map depicts the distribution of kinases co-differentially detected with TRPM7 predominant sites.

TRPM7 is a highly phosphorylated protein, with 46 autophosphorylation sites reported, 37 of which are located within Ser/Thr-rich regions positioned outside the kinase domain. These autophosphorylation sites are thought to contribute to substrate recognition and regulatory control of TRPM7 activity (Clark et al., 2008b). Beyond the autophosphorylation, no experimentally validated upstream kinases have yet been identified for TRPM7. To address this knowledge gap, we integrated kinase-substrate information from multiple kinase prediction tools and publicly available literature to infer potential upstream kinases. Based on our co-differential detection analysis, 63 kinases displayed positive co-differential detection, and 11 exhibited negative co-differential detection with the predominant TRPM7 phosphosites (Supplementary Tables S8A, B). Among the positively co-differentially detected kinases, seven kinases (PRKCD, CLK2, STK39, PKN2, MAST3, PRKD3, and MAP4K4) emerged as strong candidates for upstream regulation of TRPM7, supported by evidence from NetworKIN and AKID kinase prediction databases, as well as reports from Johnson et al. (Johnson et al., 2023) (Figure 6A). The kinome map illustrates the phylogenetic distribution of kinases that exhibit co-differential detection with TRPM7, as well as those predicted to act upstream. These candidate upstream kinases span multiple major kinase groups, including CMGC (CLK2), AGC (PRKCD, PKN2, PRKD3), STE (STK39, MAP4K4), and CAMK (MAST3) (Figure 6B). The involvement of kinases from functionally distinct pathways suggests that TRPM7 phosphorylation may be regulated by diverse signaling cascades and environmental cues. This highlights the complexity of upstream kinases of TRPM7 and prompts further investigation to validate these kinases and to explore the kinase-specific control of its biological functions.

## 4 Discussion

TRPM7 is a dual-functional protein that acts as an ion channel and as a serine/threonine kinase. As a channel, it permits the influx of divalent cations, while activation of its kinase domain enables phosphorylation and regulation of downstream effectors. Accumulating evidence indicates that dysregulation of TRPM7 contributes to multiple pathophysiological conditions, including cancer, cardiovascular disorders, and neurodegenerative diseases (Yee, 2017; Sun et al., 2015; Liu et al., 2025). However, despite its clinical importance, the functional phosphorylation sites on TRPM7, their upstream kinases, and the biological processes they regulate remain largely unexplored. To bridge this knowledge gap, we employed a systematic computational framework to analyse publicly available phosphoproteomics datasets. Through the integration of large-scale profiling and differential abundance datasets, we identified five predominant phosphorylation sites on TRPM7 that were consistently detected across diverse cellular and experimental contexts. S1255 was among the most frequently detected phosphosites, and consistent with our findings, this residue has previously been proposed as a major regulatory site in TRPM7 (Kim et al., 2012). All predominant phosphosites are located within the cytoplasmic region, specifically clustered in the serine/threonine-rich segment that precedes the C-terminal kinase domain (amino acids 1255–1513). Within the serine/threonine-rich region, S1567 has been identified as a key regulatory phosphorylation site required for TRPM7 kinase activity. (Clark et al., 2008b). These sites may play a role in regulating both the kinase and ion channel activities of TRPM7, however, their functional significance remains to be experimentally validated. Additionally, we identified several TRPM7 phosphosites that are not yet catalogued in the PhosphoSitePlus database, underscoring the need for future experimental validation of these mass spectrometry-derived sites.

Among the co-differentially detected PsOPs, TCOF1_S583 exhibited strong positive co-differential detection with TRPM7_S1255, whereas UTRN_S1405 showed negative co-differential detection. TCOF1 is a nucleolar protein essential for ribosomal RNA (rRNA) synthesis, and mutations in this gene disrupt rRNA biogenesis, leading to impaired craniofacial development (Chen et al., 2025). TRPM7 functions as a major Mg^2+^-permeable ion channel, and intracellular Mg^2+^ is a key determinant of rRNA synthesis and ribosomal stability (Pontes et al., 2016). The strong positive co-differential detection between TRPM7_S1255 and TCOF1_S583, therefore, points toward a potential functional interplay, where TRPM7-mediated Mg^2+^ homeostasis may influence TCOF1-dependent ribosome production. TCOF1 is also involved in other cellular functions, including cell cycle regulation, DND damage response, cell proliferation, and stress sensing (Grzanka and Piekielko-Witkowska, 2021). Pan-cancer expression analysis revealed elevated TCOF1 expression in cancer tissues compared to controls, and its expression was also associated with immune cell infiltration (Gu et al., 2022). In triple-negative breast cancer, increased TCOF1 expression has been reported to promote cancer progression by enhancing cancer stem cell properties (Hu et al., 2022). UTRN plays a critical role in maintaining the post-synaptic membrane and clustering of acetylcholine receptors (AChR) at neuromuscular junctions (De et al., 1997). Calcium influx is essential for the AChR-clustering activity of neural agrin. Mutations in its calcium-binding residues, which are crucial for AChR aggregation, significantly impair agrin’s ability to mediate AChR clustering (Tseng et al., 2003). Additionally, PTPN14_S593 and FKBP15_S1012 were identified as the most positively and negatively co-differentially detected phosphoproteins associated with TRPM7_S1387, respectively. PTPN14 encodes a tyrosine phosphatase that modulates multiple signaling pathways controlling cell proliferation, growth, and differentiation (Han et al., 2019). FKBP15 is involved in cytoskeletal regulation and supports the trafficking and positioning of early endosomes (Duclos et al., 2017). SRRM2_S780, OSBP_S190, and MAP4_S510 were identified as the top positively co-differentially detected phosphosites associated with TRPM7_S1477, TRPM7_S1504, and TRPM7_S1513. In contrast, DDX21_S71, AHCTF1_T1369, and MKI67_S308 represented the most strongly negatively co-differentially detected phosphosites. Notably, SRRM2 exhibited the highest number of overall phosphorylation (57 sites), among which SRRM2_S780 was more frequently detected across multiple experimental conditions. SRRM2 is a core spliceosomal component that plays a critical role in coordinating alternative mRNA splicing. Given emerging evidence demonstrating that intracellular calcium fluctuations can modulate alternative exon selection, we speculate that TRPM7-mediated calcium influx may influence splicing regulation through SRRM2 (Sharma and Lou, 2011). Further investigation is essential to determine the functional associations between TRPM7 and its co-differentially detected phosphoproteins.

Since the site-specific biological processes of most interacting proteins remain uncharacterized, we performed additional Gene Ontology analysis of all known as well as co-differentially phosphorylated interactors and substrates of TRPM7 at the protein level. This analysis of all known interacting partners revealed enrichment of broad functions such as protein phosphorylation, chromatin remodeling, intracellular signal transduction, and DNA damage response, which are consistent with the previously reported functional roles of TRPM7 (Koles et al., 2024; Cai et al., 2018; Krapivinsky et al., 2014). Beyond the enrichment of these broad biological functions, the co-differentially phosphorylated interactors and substrates were associated with more specialized processes, including positive regulation of stem cell population maintenance and regulation of mRNA splicing via the spliceosome, TGF-β signaling regulation, cell cycle G2/M transition, calcium channel regulation. Chromatin remodelling is a dynamic process driven by coordinated interactions between ATP-dependent chromatin remodelers and histone-modifying nucleosomal complexes. Any dysregulation in chromatin remodelling leads to altered gene expression and the development of diseases, including cancer (Nair and Kumar, 2012). Krapivinsky et al. (2014) demonstrated the involvement of TRPM7 in chromatin remodeling. Proteolytically cleaved fragments of TRPM7 were shown to translocate to the nucleus and interact with several components of chromatin remodeling complexes, including RYBP, DDX3X, and DDB1. In addition, TRPM7 was reported to regulate gene expression through phosphorylation of histone H3 at serine 10 (S10) and serine 28 (S28) (Krapivinsky et al., 2014). Recent findings have highlighted TRPM7’s involvement in regulating stemness through modulation of the Notch signaling pathway, thereby contributing to the growth and invasion of glioma cells (Wan et al., 2020; Liu et al., 2014), suggest that the observed enrichment of TRPM7 phosphorylation in processes related to the positive regulation of stem cell population maintenance could be significant. TRPM7 is also implicated in cell growth and proliferation by regulating cell cycle progression. Inhibition of TRPM7 using Waixenicin A has been reported to induce cell cycle arrest at the G0/G1 and G2 phases, indicating impairment of the G2/M phase transition (Zierler et al., 2011). Overall, the co-differential phosphorylation-based functional enrichment analysis could serve as a potential strategy to elucidate the functional roles of protein phosphosites. The identified TRPM7 phosphorylation-associated biological processes warrant further experimental investigation.In addition, we identified kinases, including PRKCD, CLK2, STK39, PKN2, MAST3, PRKD3, and MAP4K4, as potential upstream regulators of TRPM7. Since no upstream kinases have been characterised for TRPM7 to date, these candidates represent valuable leads for future experimental validation. Current evidence supporting the involvement of these kinases in regulating ion channel activation remains limited. However, members of the PKC family are known to modulate calcium channels, particularly voltage-dependent L-type Ca^2+^ channels (Kamp and Hell, 2000). Thus, although these kinases were identified through prediction tools, further investigation is necessary to determine whether they function as upstream regulators of TRPM7.

## 5 Conclusion

The present study serves as a valuable resource for understanding the phosphoregulatory network of TRPM7 across diverse cellular and experimental conditions. Through systematic analysis, we identified the most frequently detected phosphosites in TRPM7, highlighting their potential functional relevance. Additionally, by applying a co-differential detection analysis strategy, we inferred the possible functions of TRPM7 phosphosites based on the biological roles of its interacting partners. Moreover, we identified several candidate upstream kinases and downstream substrates for TRPM7, filling gaps in current knowledge bases. Together, these data-driven findings provide a platform for future studies to experimentally validate TRPM7-centred regulatory mechanisms and further elucidate its roles as a therapeutic target for cancer.

## 6 Limitations

The present study is based on retrospective analysis of publicly available phosphoproteomics datasets and is therefore subject to several inherent limitations. Integration of multiple studies may introduce variability arising from differences in experimental design, sample types, and detection sensitivity. Although stringent inclusion criteria and multi-level filtering approaches were applied to enhance data reliability, technical variability, batch effects, and biases due to diverse experimental conditions cannot be completely ruled out. The functional interpretation of TRPM7 was primarily derived from frequently detected phosphosites across datasets, which may lead to underrepresentation of low-abundance or context-specific phosphorylation events that could also play important biological roles. In addition, the inference from this study is based on computational analyses and therefore requires further validation. Experimental validation in relevant biological systems, including clinical samples and animal models, is required to confirm these findings. Future studies employing approaches such as site-directed mutagenesis, kinase assays, and CRISPR/Cas9-mediated phosphosite editing will be important to validate the proposed upstream kinases and downstream substrates. Furthermore, phospho-specific antibody-based analyses will be necessary to assess the expression and clinical relevance of key TRPM7 phosphosites across different model systems.

Despite these limitations, this study provides a systematic and data-driven framework to explore the functional roles of TRPM7 phosphosites and their regulatory networks. The findings offer a set of testable hypotheses and a useful resource for guiding future experimental investigations.

## Data Availability

The original contributions presented in the study are included in the article/Supplementary Material, further inquiries can be directed to the corresponding author.
